# Editorial

**DOI:** 10.2478/v10102-010-0007-1

**Published:** 2010-06

**Authors:** Mojmír Mach

**Affiliations:** Institute of Experimental Pharmacology & Toxicology, Dúbravská cesta Bratislava, Slovakia

The year 2010 is not gone yet but we already know that this year will be associated with one of the biggest losses in pharmacology and toxicology. On Tuesday, April 13, 2010, at the age of 97, one of the most influential pharmacologists in Eastern Europe, Professor Helena Rašková has left us. Her wisdom, generosity, kindness and hard work made it possible to create a positive environment for research and education, although sometimes she had to fight hard. Her incredible story „How I became a Pharmacologist” wrote by Prof. Rašková herself documents how rich and inspirational her life was [Rašková H. (1997). *Pharmacology & Toxicology* **80**: 255–261].

In this issue you will find mostly works of her colleagues and friends who would like to pay homage to the mother and grandmother of Czecho-Slovak pharmacology and toxicology and to the wonderful person who Prof. Helena Rašková was. She touched many people in her lifetime, affected many and will truly be missed.

